# Acquisition of peak bone mass in a Norwegian youth cohort: longitudinal findings from the Fit Futures study 2010–2022

**DOI:** 10.1007/s11657-024-01414-2

**Published:** 2024-07-03

**Authors:** Edvard H. Sagelv, Nina Emaus, Elin Evensen, Tore Christoffersen, Elaine Dennison, Anne-Sofie Furberg, Guri Grimnes, Jonas Johansson, Christopher Sivert Nielsen, Ole Andreas Nilsen, Anne Winther

**Affiliations:** 1 Division of Neurosciences, Orthopedics and Rehabilitation Services, University Hospital of North Norway, Tromsø, Norway; 2 Department of Health and Care Sciences, Faculty of Health Sciences, UiT the Arctic University of Norway, Tromsø, Norway; 3 School of Sports Sciences, Faculty of Health Sciences, UiT the Arctic University of Norway, Alta, Norway; 4 Finnmark Hospital Trust, Alta, Norway; 5 MRC, Lifecourse Epidemiology Centre, University of Southampton, Southampton, UK; 6 Department of Microbiology and Infection Control, University Hospital of North Norway, Tromsø, Norway; 7 Department of Health and Social Sciences, Molde University College, Molde, Norway; 8 Department of Clinical Medicine, Faculty of Health Sciences, UiT the Arctic University of Norway, Tromsø, Norway; 9 Division of Medicine, University Hospital of North Norway, Tromsø, Norway; 10 Department of Community Medicine, Faculty of Health Sciences, UiT The Arctic University of Norway, Tromsø, Norway; 11 Department of Chronic Diseases, Norwegian Institute of Public Health, Oslo, Norway; 12 Department of Pain Management and Research, Oslo University Hospital, Oslo, Norway

**Keywords:** Bone mineral density, Peak bone mass, Adolescents, Young adulthood, Population-based study

## Abstract

***Summary*:**

In a Norwegian youth cohort followed from adolescence to young adulthood, bone mineral density (BMD) levels declined at the femoral neck and total hip from 16 to 27 years but continued to increase at the total body indicating a site-specific attainment of peak bone mass.

**Purpose:**

To examine longitudinal trends in bone mineral density (BMD) levels in Norwegian adolescents into young adulthood.

**Method:**

In a prospective cohort design, we followed 980 adolescents (473 (48%) females) aged 16–19 years into adulthood (age of 26–29) on three occasions: 2010–2011 (Fit Futures 1 (FF1)), 2012–2013 (FF2), and 2021–2022 (FF3), measuring BMD (g/cm^2^) at the femoral neck, total hip, and total body with dual x-ray absorptiometry (DXA). We used linear mixed models to examine longitudinal BMD changes from FF1 to FF3.

**Results:**

From the median age of 16 years (FF1), femoral neck BMD (mean g/cm^2^ (95% CI)) slightly increased in females from 1.070 (1.059–1.082) to 1.076 (1.065–1.088, *p* = 0.015) at the median age of 18 years (FF2) but declined to 1.041 (1.029–1.053, *p* < 0.001) at the median age of 27 years (FF3). Similar patterns were observed in males: 16 years, 1.104 (1.091–1.116); 27 years, 1.063 (1.050–1.077, *p* < 0.001); and for the total hip in both sexes (both *p* < 0.001). Total body BMD increased from age 16 to 27 years in both sexes (females: 16 years, 1.141 (1.133–1.148); 27 years, 1.204 (1.196–1.212), *p* < 0.001; males: 16 years, 1.179 (1.170–1.188); 27 years, 1.310 (1.296–1.315), *p* < 0.001).

**Conclusion:**

BMD levels increased from 16 to 18 years at the femoral and total hip sites in young Norwegian females and males, and a small decline was observed at the femoral sites when the participants were followed up to 27 years. Total body BMD continued to increase from adolescence to young adulthood.

**Supplementary Information:**

The online version contains supplementary material available at 10.1007/s11657-024-01414-2.

## Introduction

The incremental societal burden of osteoporotic fractures in the elderly is tremendous [1]. In Norway, the country with the highest reported fracture incidences worldwide [2–6], hip fracture rates have declined over a 15-year period between 1999 and 2013 [7, 8]. However, due to the aging population, the total number is expected to rise [7]. In addition to the added burden due to other osteoporotic fractures [9], hip fractures remain a serious health burden in Norway today [10].

Osteoporotic fractures mainly occur in adults over 50 years, and early preventive strategies are advocated to identify high-risk individuals and to reduce the burden of osteoporotic fractures [11–13]. Although age and sex contribute to 10-year fracture risk estimates independently of bone mineral density (BMD) [14–16], BMD constitutes a central part of the definition and diagnosis of osteoporosis [17, 18]. From a lifetime perspective, premenopausal bone mass maintenance in women and peak bone mass (PBM) attainment in adolescence are important predictors of future fracture risk [19].

One simulation study estimated that a 10% increase in PBM may delay the development of osteoporosis by 13 years [20]. This delay is greater than the 2 years found to be obtained by maximizing BMD levels following menopause or by slowing menopausal bone loss rates [20]. Similar findings are reported by others [21–23], indicating that the promotion of bone health in youth, before the onset of bone loss, is important to further combat future osteoporosis and fractures [23].

Previous research has indicated that femoral neck and total hip PBM are achieved at ~ 15 to 19 years in females and ~ 16 to 19 years in males [19, 24–27]. For lumbar spine, bone mass appears to peak later in life, between 33 and 40 years in females and between 19 and 33 years in males [24]. Similar findings were observed for forearm BMD, where PBM were achieved between 30 and 40 years [28]. This indicates that PBM attainment is site-specific.

In the present study, we aimed to examine the longitudinal trends in BMD at the femoral neck, total hip, and total body from adolescence at 16–19 years to young adulthood at 26–29 years in a Norwegian youth cohort. Secondary aims were to compare the BMD levels of Norwegian adolescents with the Lunar reference data from adolescence to young adulthood.

## Methods

### Study population

**Fig. 1:**
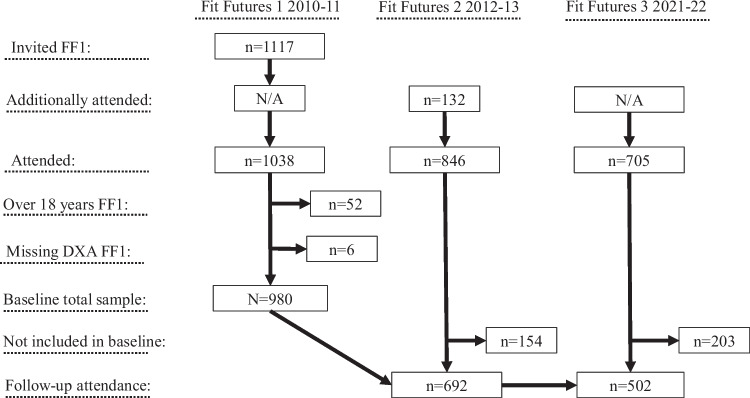
Flow chart of the included participants

We included adolescents attending the Fit Futures study (FF) [29] in a prospective cohort design. The FF includes three waves of data collection: FF1, 2010–2011; FF2, 2012–2013; and FF3, 2021–2022. All first-year students (*n* = 1117) in all upper-secondary schools in Tromsø and Balsfjord municipalities, North Norway, were invited to participate in FF1 and 1038 students (92.9%) attended [30]. All who attended FF1 were invited to the follow-up surveys, of which 714 (68.8%) attended FF2 and 642 (61.8%) attended FF3. Additionally, 132 new upper-secondary students attended FF2, leaving total sample sizes at 846 in FF2 and 705 in FF3. We included those under 19 who underwent dual x-ray absorptiometry (DXA) scans at baseline (FF1). Of the 1038 participants who attended at baseline (FF1), 52 were 19 years or older, and 6 were missing total body DXA scans. Therefore, we ended up with a sample of 980 participants, of which 473 were females and 507 males. From this cohort, 692 (females, *n* = 381; males, *n* = 311) and 502 (females, *n* = 281; males, *n* = 221) attended FF2 and FF3, respectively. A flow chart of included participants is found in Fig. 1. In total, 462 (females, *n* = 251; males, *n* = 211) attended all surveys and provided valid DXA scans at all measurement time points (not shown in flow as this is not the total sample size).

### Ethics

The present study was approved by the Regional Committee of Medical Research Ethics (ref. 2013/1459/ REK Nord). The Fit Futures study is a population-based health survey and has since 2018 been regulated by the Regulations on population-based health research and the Data Protection Legislation in Norway. The participants have given written informed consent at all three waves. Participants below 16 years of age in FF1 had to bring additional written consent from a legal guardian to attend the survey.

### Measurements of BMD

In all three waves of FF, BMD (g/cm^2^) was measured with DXA (GE Lunar Prodigy, Lunar Corporation, USA), the gold standard for BMD measurements [31], at the femoral neck, the total hip, and total body. DXA scans were performed by trained technicians, and quality assessment procedures were performed according to protocol on a daily basis. The coefficient of variation of the DXA machine used in this study has previously been estimated to be 1.17% for the total hip and 1.72% for femoral neck measurements [32]. The total body coefficient of variation has not been estimated for the DXA machine used in this study. *Z*-scores were calculated according to the Lunar reference population, whose reference data derives from multiple cohort studies (total *n* = 2818 participants) [33–38]; *z*-scores reported in this study are calculated by age, sex, and ethnicity using appropriate age- and sex-matched reference data for adolescents under 20 years (FF1 and FF2) and young adults between 20 and 29 years (FF3).

### Descriptive data

In all surveys, height and weight were measured in all participants according to the same protocol to the nearest 0.1 cm and 0.1 kg on an automatic electronic scale (Jenix DS 102 stadiometer, Dong Sahn Jenix, Seoul, Korea) wearing light clothing and no shoes. We calculated the body mass index (BMI) as weight divided by height squared (kg/m^2^) and used iso-BMI to classify normal weight (corresponding to < 25 kg/m^2^ for adults), overweight (25–29 kg/m^2^ for adults), and obese (> 30 kg/m^2^ for adults) participants according to Cole and Lobstein [39]. We measured fat mass (kg) and lean mass (kg) using total body DXA scans. To determine puberty, girls were asked if and when they had started menarche, and boys were rated according to the Pubertal Development Score by Paterson et al. [40], ranging from 1 to 4. Alcohol intake (frequency of drinking), leisure time physical activity (Saltin–Grimby physical activity scale [41]), screen time (mean week and weekend hours per day), smoking (never, sometimes, daily), snuff (never, sometimes, daily), high school study program (general, sport, and vocational training), self-perceived health (very bad, bad, neither good nor bad, good, excellent), vitamin (daily, sometimes, never), cod liver oil (daily, sometimes, never), milk intake (frequency), and cheese intake (frequency) were obtained from questionnaires.

### Statistical analyses

To compare differences in baseline characteristics between those attending all three surveys and those only attending FF1 or FF1 and FF2, we used independent sample *t*-tests (weight, BMI, fat mass, lean mass, and femoral neck, total hip, and total body BMD) and Pearson’s chi-square tests (physical activity level). We used linear mixed models with maximum likelihood and a random intercept on the subject level to test the main effect of time (surveys 1, 2, and 3) for changes in BMD and *z*-score (Lunar reference data) at the femoral neck, total hip, and total body. To compare changes from surveys 1 to 2 and 3, we modeled time (survey) as a categorical variable. We also ran linear mixed models adjusted for weight to examine whether weight was a confounding source of BMD changes. The covariance structure was set to first-order autoregressive (AR1) with homogeneous variance. All analyses were stratified by sex. For sensitivity analysis, we used repeated measures univariate analysis of variance (ANOVA) to examine the longitudinal changes in BMD levels by only including those participating in FF1, FF2, and FF3 with valid DXA scans (females, *n* = 251; males, *n* = 211). Data are shown as mean with 95% confidence intervals (CI) and as mean ± standard deviation (SD) and frequency (%) for descriptive values. All statistical analyses were performed using Stata version 17 (StataCorp LLC, TX, USA).

## Results

The majority of the participants were 16 years old (females, 16.2 ± 0.5; males, 16.1 ± 0.6), normal weight, and attended general studies at baseline (Table 1). About 70% rated their health as good or excellent, and most had never smoked or used snuff (Table 1). Most of them were physically active in their leisure time and drank alcohol less than once per week (Table 1). At baseline, females attending all three surveys had higher weight (*p* = 0.041), more lean mass (*p* < 0.001), and higher BMD levels (all *p* < 0.03) than females only attending FF1 or FF1 and FF2 (Supplementary Table S1). There were no differences in baseline characteristics between males attending all three surveys versus males only attending FF1 or FF1 and FF2 (Supplementary Table S1).

**Table 1:** Descriptive characteristics at baseline. The Fit Futures 1 2010–2011

	Females	Males
Total, *n*	473	507
Age (yrs), mean ± SD	16.2 ± 0.5	16.1 ± 0.6
15 years,* n* (%)	14 (3.0)	36 (7.1)
16 years,* n* (%)	380 (80.3)	390 (76.9)
17 years,* n* (%)	72 (15.2)	65 (12.8)
18 years,* n* (%)	7 (1.5)	16 (3.2)
Anthropometric, *n*	473	507
Height (m), mean ± SD	1.65 ± 0.07	1.77 ± 0.07
Weight (kg), mean ± SD	61.0 ± 11.5	70.3 ± 14.4
BMI (kg/m^2^), mean ± SD	22.5 ± 4.0	22.4 ± 4.2
Normal weight (< 25 kg/m^2^)*	368 (77.8)	376 (74.2)
Overweight (25–29 kg/m^2^)*	76 (16.1)	92 (18.1)
Obese (≥ 30 kg/m^2^)*	29 (6.1)	39 (7.7)
Dual x-ray scan,* n*	473	507
Fat mass (kg), mean ± SD	20.5 ± 6.2	14.7 ± 10.8
Lean mass (kg), mean ± SD	38.5 ± 4.6	53.7 ± 6.9
High school main program,* n*	473	507
General studies,* n* (%)	242 (51.2)	148 (29.2)
Sports high school,* n* (%)	38 (8.0)	65 (12.8)
Vocational training,* n* (%)	193 (40.8)	294 (58.0)
Puberty,* n*	470	398
Menarche girls,* n* (%)	467 (99.4)	N/A
Menarche age (year), mean ± SD	12.7 ± 1.2	N/A
PDS boys, *n* (%)^#^	N/A	3.3 ± 0.4
Self-perceived health,* n*	380	320
Very bad,* n* (%)	0 (0)	3 (0.9)
Bad,* n* (%)	17 (4.5)	14 (4.4)
Neither good nor bad,* n* (%)	74 (19.5)	73 (22.8)
Good,* n* (%)	206 (54.2)	139 (43.4)
Excellent,* n* (%)	83 (21.8)	91 (28.4)
Smoking,* n*	468	499
Never,* n* (%)	373 (79.9)	379 (76.0)
Sometimes,* n* (%)	77 (16.5)	103 (20.6)
Daily,* n* (%)	18 (3.9)	17 (3.4)
Snuff,* n*	469	498
Never,* n* (%)	310 (66.1)	293 (58.8)
Sometimes,* n* (%)	67 (14.3)	64 (12.9)
Daily,* n* (%)	92 (19.6)	141 (28.3)
Alcohol frequency,* n*	474	498
Never,* n* (%)	111 (23.4)	159 (31.9)
Once per month or less,* n* (%)	219 (46.2)	185 (37.2)
2–4 times per month,* n* (%)	136 (28.7)	145 (29.1)
2–3 times per week,* n* (%)	8 (1.7)	6 (1.2)
4 or more times per week,* n* (%)	0 (0)	3 (0.6)
Leisure time physical activity, *n*	470	499
Inactive,* n* (%)	65 (13.8)	148 (29.7)
Moderate,* n* (%)	191 (40.6)	125 (25.1)
Vigorous,* n* (%)	137 (29.2)	114 (22.9)
Very vigorous,* n* (%)	77 (16.4)	112 (22.4)
Screen time,* n* (%)	464	498
Hours∙week^−1^, mean ± SD	6.3 ± 1.4	6.9 ± 1.4
Vitamin supplements, *n*	492	516
Daily,* n* (%)	161 (32.7)	215 (41.7)
Sometimes,* n* (%)	220 (44.7)	211 (40.9)
Never,* n* (%)	111 (22.6)	90 (17.4)
Cod liver oil supplement, *n*	492	516
Daily,* n* (%)	233 (47.4)	259 (50.2)
Sometimes,* n* (%)	166 (33.7)	174 (33.7)
Never,* n* (%)	93 (18.9)	83 (16.1)
Milk intake, *n*	486	513
Glass∙day^−1^, mean ± SD	2.0 ± 1.8	2.5 ± 2.4
No glasses,* n* (%)	55 (11.3)	59 (11.5)
1–2 glasses∙day^−1^,* n* (%)	261 (53.7)	231 (45.0)
2.1–4 glasses∙day^−1^,* n* (%)	124 (25.5)	126 (24.6)
> 4 glasses∙day^−1^,* n* (%)	46 (9.5)	97 (18.9)
Cheese intake, *n*	495	515
Servings∙week^−1^, mean ± SD	2.5 ± 1.9	2.8 ± 2.0
Never,* n* (%)	27 (5.5)	25 (4.9)
1 serving∙week^−1^,* n* (%)	88 (17.6)	76 (14.8)
2 servings∙week^−1^,* n* (%)	204 (41.2)	194 (37.7)
5 servings∙week^−1^,* n* (%)	177 (35.8)	220 (42.7)

Data are shown as mean ± SD or as frequency (%)

*n* number of participants with information, *BMI* body mass index, *PDS* puberty development score, *SD* standard deviation

^*^Iso-BMI derived from Cole and Lobstein, 2012, *Pediatr Obes*[39]

^#^PDS score from Petersen et al., 1988, *J Youth Adolesc*[40]

**Fig. 2:**
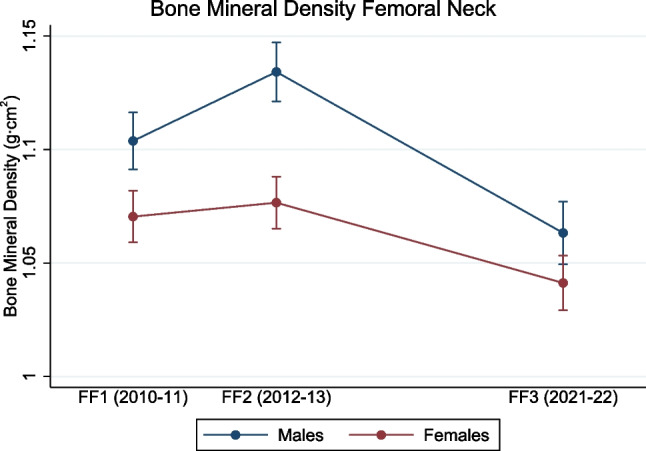
Longitudinal changes in bone mineral density at the femoral neck (the Fit Futures study 2010–2022). Data are shown as mean with error bars as 95% confidence intervals

We observed a main effect of time in femoral neck BMD acquisition for both females and males (both *p* < 0.001) (Fig. 2). In females, the femoral neck BMD slightly increased from 1.070 g/cm^2^ (95% CI, 1.059–1.082 g/cm^2^) in FF1 to 1.076 g/cm^2^ (95% CI, 1.065–1.088 g/cm^2^; *p* = 0.015) in FF2 but thereafter declined to 1.041 g/cm^2^ (95% CI, 1.029–1.053 g/cm^2^; *p* < 0.001) in FF3 (Fig. 2). A similar pattern was observed in males, where the femoral neck BMD increased from 1.104 g/cm^2^ (95% CI, 1.091–1.116 g/cm^2^) in FF1 to 1.134 g/cm^2^ (95% CI, 1.121–1.147 g/cm^2^; *p* < 0.001) in FF2 and declined to 1.063 g/cm^2^ (95% CI, 1.050–1.077 g/cm^2^; *p* < 0.001) in FF3 (Fig. 2).

**Fig. 3:**
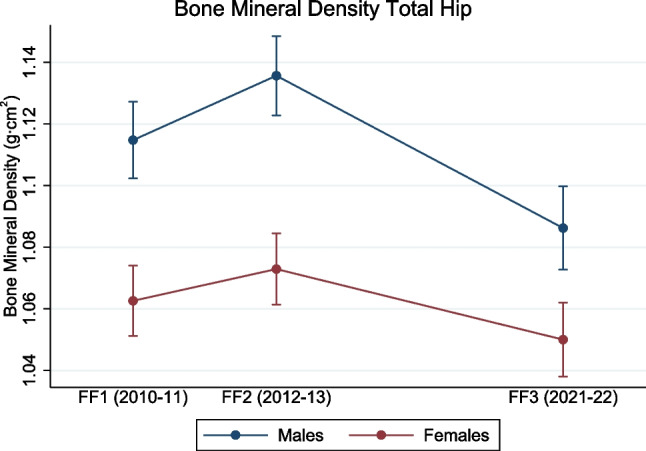
Longitudinal changes in bone mineral density at the total hip (the Fit Futures study 2010–2022). Data are shown as mean with error bars as 95% confidence intervals

Similar patterns were observed for total hip BMD in both sexes (main effect of time, both *p* < 0.001) (Fig. 3). In females, the total hip BMD increased from 1.062 g/cm^2^ (95% CI, 1.051–1.074 g/cm^2^) in FF1 to 1.073 g/cm^2^ (95% CI, 1.061–1.084 g/cm^2^; *p* < 0.001) in FF2, with a decline to 1.050 g/cm^2^ (95% CI, 1.038–1.062 g/cm^2^; *p* < 0.001) in FF3 (Fig. 3). In males, the total hip BMD increased from 1.115 g/cm^2^ (95% CI, 1.102–1.127 g/cm^2^) in FF1 to 1.136 g/cm^2^ (95% CI, 1.123–1.149 g/cm^2^; *p* < 0.001) in FF2 and decreased to 1.086 g/cm^2^ (95% CI, 1.072–1.100 g/cm^2^; *p* < 0.001) in FF3 (Fig. 3).

**Fig. 4:**
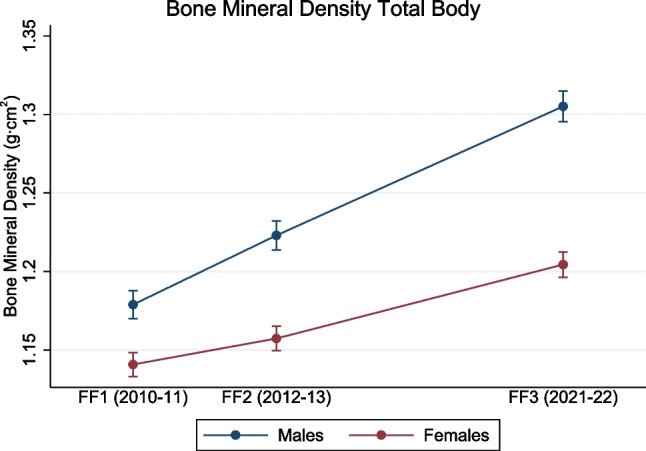
Longitudinal changes in bone mineral density for total body (the Fit Futures study 2010–2022). Data are shown as mean with error bars as 95% confidence intervals

The total body BMD steadily increased in both females and males from FF1 to FF3 (main effect of time, both *p* < 0.001) (Fig. 4). In females, the total body BMD increased from 1.141 g/cm^2^ (95% CI, 1.133–1.148 g/cm^2^) in FF1 to 1.157 g/cm^2^ (95% CI, 1.150–1.165 g/cm^2^; *p* < 0.001) in FF2 to 1.204 g/cm^2^ (95% CI, 1.196–1.212 g/cm^2^) in FF3 (*p* < 0.001) (Fig. 4). In males, the total body BMD increased from 1.179 g/cm^2^ (95% CI, 1.170–1.188 g/cm^2^) in FF1 to 1.222 g/cm^2^ (95% CI, 1.213–1.232 g/cm^2^, *p* < 0.001) in FF2 to 1.310 g/cm^2^ (95% CI, 1.296–1.315 g/cm^2^) in FF3 (*p* < 0.001) (Fig. 4).

In models adjusted for weight, patterns of associations with time were generally similar to the unadjusted models (all main effects of time, *p* < 0.001) (Supplementary Table S2). However, females did not change their femoral neck BMD from FF1 to FF2 (*p* = 0.74) (Table 2) as observed in the unadjusted model (Fig. 2).

**Table 2:** The longitudinal change in *z*-score in girls and boys. The Fit Futures 2010–2022

		Fit Futures 1 (2010–2011)	Fit Futures 2 (2012–2013)	Fit Futures 3 (2021–2022)	Main effect of time
Femoral neck					
Girls (*n* = 474)	Mean (95% CI)	0.51 (0.43 to 0.59)	0.60 (0.50 to 0.70)	− 0.001 (− 0.10 to 0.10)	< 0.001
Boys (*n* = 504)	Mean (95% CI)	0.11 (0.02 to 0.21)	0.18 (0.08 to 0.28)	− 0.23 (− 0.33 to − 0.13)	< 0.001
Total hip					
Girls (*n* = 474)	Mean (95% CI)	0.39 (0.30 to 0.48)	0.48 (0.40 to 0.57)	0.23 (0.14 to 0.32)	< 0.001
Boys (*n* = 504)	Mean (95% CI)	0.13 (0.04 to 0.22)	0.11 (0.02 to 0.21)	− 0.15 (− 0.25 to − 0.06)	< 0.001
Total body					
Girls (*n* = 476)	Mean (95% CI)	0.20 (0.11 to 0.29)	0.48 (0.38 to 0.56)	0.97 (0.88 to 1.07)	< 0.001
Boys (*n* = 507)	Mean (95% CI)	0.28 (0.19 to 0.36)	0.02 (− 0.07 to 0.11)	0.87 (0.77 to 0.96)	< 0.001

Data are shown as mean *z*-scores and 95% CI

Main effect of time from the linear mixed model

*CI* confidence intervals

When comparing the FF sample with the Lunar Prodigy reference database, *z*-scores for the femoral neck were unchanged from FF1 to FF2 in females (*p* = 0.17) but decreased to below zero in FF3 (*p* < 0.001) (Table 2). In males, femoral neck *z*-scores increased from FF1 to FF2 (*p* = 0.02) and decreased to below zero in FF3 (*p* < 0.001) (Table 2). Total hip *z*-scores in females increased from FF1 to FF2 (*p* < 0.001) and decreased to FF3 (*p* < 0.001). In males, the total hip *z*-score was unchanged from FF1 to FF2 (*p* = 0.48) and decreased to below zero in FF3 (*p* < 0.001) (Table 2). Total body *z*-scores were positive and increased from FF1 to FF3 for both females and males (all *p* < 0.001) (Table 2).

In sensitivity analysis only including those participating in all surveys, the results remained unchanged compared with the main analysis (Supplementary Table S3).

## Discussion

In this Norwegian youth cohort followed over 10 years from the median of 16 to 27 years, PBM levels at the femoral neck and total hip seemed to be reached in the second decade since BMD increased from 16 to 19 years but decreased up to the median age of 27. Total body BMD levels continued with a steady increase from adolescence to young adulthood. These patterns were mirrored when comparing *z*-scores for the Lunar reference database.

Our observation of attained femoral neck and total hip BMD levels in the second decade is consistent with previous research [19, 24]. Notably, males had a greater incline in BMD levels at the femoral neck and total hip than females from FF1 to FF2, which suggests that males reach peak BMD levels later than females. This has also been observed in previous studies. In one study, peak femoral neck BMD was observed at 19 years among 1052 males aged 18–28 years [42], and in another study, femoral neck and total hip BMD peaked at 19–21 years for males and 16–19 years for females [24].

However, we also observed that peak BMD levels are site-specific, where total body BMD increased from FF1 to FF3. The site-specific difference in BMD for the femoral neck and total hip at 16–19 years as compared to the total body BMD later in life was also observed previously [24–27, 43]. In a previous study, lumbar spine BMD levels increased from 16 to 32 years [24], and in another study, forearm PBM was reached between the ages of 30 and 40 years [28]. Thus, it is plausible that we did not observe peak total body BMD when participants were ~ 27 years old and that it may further increase, potentially towards 30 years [43].

We observed that the positive *z*-scores at the femoral neck and total hip for females and males in FF1 and FF2 turned negative at FF3, except at the total hip in females where all three measurement points of *z*-scores were positive. Previous studies in the same Norwegian adolescent sample as used in our study showed that BMD levels in Norwegian adolescents between 16 and 18 years appeared to be slightly higher than the Lunar pediatric reference data [30]. However, in this study, the femoral neck and total hip BMD *z*-scores approximated the Lunar reference data when participants were 27 years old, indicating that in the transition to young adulthood, an observed advantage at the median age of 16 to 18 years [30] is no longer present at adult age. The reasons for this shift are not easily explainable. However, we cannot rule out a possible cohort effect. For example, some participants studied sports during high school, which may indicate them being very active during adolescence, while they may perform similar physical activity levels in young adulthood; however, this is only speculation from our side. Other lifestyle and environmental factors may also contribute to this development and warrant further investigation.

At the same time, total body BMD *z*-scores were increasing with increasing age, which may be promising for lowering the fracture risk in general [44]. Nevertheless, more research on the reasons for the observed decline in the femoral neck and total hip BMD levels in the transition to young adulthood, and whether total and upper body BMD levels contribute to lower future fracture risk, is warranted.

### Strengths

The strength of the present study is the population-based design within a region with an identified high risk of osteoporotic fractures. Moreover, our study had high attendance rates throughout the surveys, with 93% in the baseline survey in 2010–2011 and 68.8% and 61.8% in the follow-up surveys, respectively. Although potential selection bias cannot be ruled out, especially in follow-up surveys where those being healthier potentially agreed to participate, we used linear mixed models that utilize all available data to compensate for dropouts over time. Moreover, our DXA data was derived from trained technicians conducting strict quality control on densitometer performance, which likely secured BMD measurements with high precision [32].

### Limitations

As we only had access to two-dimensional DXA measurements, which is a surrogate determinant of bone strength [45], we lacked an opportunity to evaluate the development of cancellous and cortical bone compartments or to capture changes in the microarchitecture of the bones [22, 23]. Bone growth during maturation involves both accumulations of bone mass and expansion of bone volume, and these two processes do not always occur in parallel [46]. Indeed, BMD values should be interpreted with care in individuals with a growing skeleton, as skeletal strength may increase due to an increased area despite decreasing BMD [42]. Consequently, this may have influenced our interpretation of the longitudinal trends.

## Conclusion

In this prospective cohort study, BMD levels increased from 16 to 18 years at the femoral and total hip sites in young Norwegian females and males, and a small decline in BMD was observed at the femoral sites in the third decade when participants were 27 years old. Total body BMD increased from adolescence to young adulthood. More studies on longitudinal trends are warranted to validate whether similar declines in the femoral neck and total hip BMD levels in this age span are observed elsewhere.

## Supplementary Information

Below is the link to the electronic supplementary material.

Supplementary file1 (DOCX 17 KB)

Supplementary file2 (DOCX 16 KB)

Supplementary file3 (DOCX 17 KB)

## Data Availability

The data underlying this study were provided by the Fit Futures under license and so are not publicly available. Data can be shared from Fit Futures upon application to the Fit Futures data and publication committee: fit.futures@uit.no.
