# Supplementary material for: Acquisition of peak bone mass in a Norwegian youth cohort: longitudinal findings from the Fit Futures study 2010–2022
Source: Arch Osteoporos. 2024 Jul 3;19(1):58. doi: 10.1007/s11657-024-01414-2 (PMC11222189; doi:10.1007/s11657-024-01414-2)
Supplement: Supplementary file 1 — Supplementary file1 (DOCX 17 KB) [file 11657_2024_1414_MOESM1_ESM.docx]

**Supplementary Table S1.** Comparison of those attending all surveys versus those only attending Fit Futures 1 or those attending Fit Futures 1 and 2. The Fit Futures 2010-2022.

|  | **Only attending Fit futures 1** | **Attending all surveys** | **P_equiality_** |
| --- | --- | --- | --- |
| *Females (n)* | *89* | *272* |  |
| **Weight (kg),** mean ± SD | 58.4 ± 10.6 | 61.1 ± 10.5 | 0.041 |
| **BMI (kg/m^2^),** mean ± SD | 21.9 ± 3.8 | 22.3 ± 3.8 | 0.44 |
| **Fat mass (kg),** mean ± SD | 19.4 ± 8.1 | 20.0 ± 8.3 | 0.58 |
| **Lean mass (kg),** mean ± SD | 37.0 ± 4.3 | 39.1 ± 4.4 | <0.001 |
| **Physical Activity** |  |  | 0.14 |
| Inactive, n (%) | 14 (15.7) | 31 (11.4) |  |
| Moderately Active, n (%) | 39 (43.8) | 103 (37.9) |  |
| Vigorously Active, n (%) | 27 (30.3) | 83 (30.5) |  |
| Very vigorously Active, n (%) | 9 (10.1) | 55 (20.2) |  |
| **BMD femoral neck (g/cm^2^),** mean ± SD | 1.047 ± 0.117 | 1.081 ± 0.126 | 0.027 |
| **BMD total hip (g/cm^2^),** mean ± SD | 1.037 ± 0.113 | 1.074 ± 0.127 | 0.016 |
| **BMD total body (g/cm^2^),** mean ± SD | 1.122 ± 0.072 | 1.145 ± 0.075 | 0.011 |
| *Males (n)* | *96* | *211* |  |
| **Weight (kg),** mean ± SD | 71.9 ± 13.9 | 69.2 ± 13.7 | 0.12 |
| **BMI (kg/m^2^),** mean ± SD | 22.7 ± 4.1 | 2.1 ± 3.9 | 0.21 |
| **Fat mass (kg),** mean ± SD | 15.9 ± 11.0 | 13.8 ± 10.4 | 0.12 |
| **Lean mass (kg),** mean ± SD | 54.4 ± 6.8 | 53.7 ± 6.6 | 0.34 |
| **Physical Activity** |  |  | 0.32 |
| Inactive, n (%) | 30 (31.3) | 52 (25.0) |  |
| Moderately Active, n (%) | 19 (19.8) | 59 (28.4) |  |
| Vigorously Active, n (%) | 22 (22.9) | 52 (25.0) |  |
| Very vigorously Active, n (%) | 25 (26.0) | 45 (21.6) |  |
| **BMD femoral neck (g/cm^2^),** mean ± SD | 1.106 ± 0.145 | 1.117 ± 0.147 | 0.54 |
| **BMD total hip (g/cm^2^),** mean ± SD | 1.114 ± 0.153 | 1.127 ± 0.144 | 0.47 |
| **BMD total body (g/cm^2^),** mean ± SD | 1.181 ± 0.094 | 1.181 ± 0.095 | 0.98 |

Data are shown as mean ± SD or frequency (%). SD=standard deviation. P_equiality_=p-value from independent sample t-tests or Pearson´s Chi Square.
