# Supplementary material for: Acquisition of peak bone mass in a Norwegian youth cohort: longitudinal findings from the Fit Futures study 2010–2022
Source: Arch Osteoporos. 2024 Jul 3;19(1):58. doi: 10.1007/s11657-024-01414-2 (PMC11222189; doi:10.1007/s11657-024-01414-2)
Supplement: Supplementary file 2 — Supplementary file2 (DOCX 16 KB) [file 11657_2024_1414_MOESM2_ESM.docx]

**Supplementary Table S2.** The longitudinal change in bone mineral density in females and males adjusted for weight. The Fit Futures 2010-2022.

|  |  | **Fit Futures 1**  **(2010-11)** | **Fit Futures 2**  **(2012-13)** | **Fit Futures 3**  **(2021-22)** | **Main effect of time** |
| --- | --- | --- | --- | --- | --- |
| **Femoral neck** |  |  |  |  |  |
| Females  g/cm^2^ | Mean  (95%CI) | 1.077  1.067-1.088 | 1.078  1.067-1.089 | 1.028  1.016-1.040 | <0.001 |
| Males  g/cm^2^ | Mean  (95%CI) | 1.117  1.105-1.129 | 1.133  1.121-1.145 | 1.038  1.025-1.052 | <0.001 |
| **Total hip** |  |  |  |  |  |
| Females  g/cm^2^ | Mean  (95%CI) | 1.071  1.060-1.081 | 1.075  1.064-1.086 | 1.035  1.024-1.047 | <0.001 |
| Males  g/cm^2^ | Mean  (95%CI) | 1.127  1.115-1.139 | 1.135  1.123-1.147 | 1.064  1.050-1.077 | <0.001 |
| **Total body** |  |  |  |  |  |
| Females  g/cm^2^ | Mean  (95%CI) | 1.147  1.140-1.154 | 1.158  1.152-1.165 | 1.193  1.185-1.200 | <0.001 |
| Males  g/cm^2^ | Mean  (95%CI) | 1.193  1.185-1.200 | 1.222  1.214-1.230 | 1.279  1.270-1.288 | <0.001 |

Data are shown as mean and 95%CI adjusted for weight. CI=confidence intervals. Main effect of time from the Linear mixed model.
