# Supplementary material for: Acquisition of peak bone mass in a Norwegian youth cohort: longitudinal findings from the Fit Futures study 2010–2022
Source: Arch Osteoporos. 2024 Jul 3;19(1):58. doi: 10.1007/s11657-024-01414-2 (PMC11222189; doi:10.1007/s11657-024-01414-2)
Supplement: Supplementary file 3 — Supplementary file3 (DOCX 17 KB) [file 11657_2024_1414_MOESM3_ESM.docx]

**Supplementary Table S3.** The longitudinal change in bone mineral density using repeated measures ANOVA. The Fit Futures 2010-2022.

|  |  | **Fit Futures 1**  **(2010-11)** | **Fit Futures 2**  **(2012-13)** | **Fit Futures 3**  **(2021-22)** | **Main effect of time** |
| --- | --- | --- | --- | --- | --- |
| **Femoral neck** |  |  |  |  |  |
| Females  g/cm^2^ | Mean  (95%CI) | 1.076  1.071-1.081 | 1.082  1.079-1.088 | 1.049  1.044-1.054 | <0.001 |
| Males  g/cm^2^ | Mean  (95%CI) | 1.112  1.108-1.123 | 1.147  1.140-1.154 | 1.078  1.071-1.085 | <0.001 |
| **Total hip** |  |  |  |  |  |
| Females  g/cm^2^ | Mean  (95%CI) | 1.072  1.067-1.076 | 1.082  1.077-1.086 | 1.060  1.055-1.065 | <0.001 |
| Males  g/cm^2^ | Mean  (95%CI) | 1.127  1.120-1.134 | 1.148  1.142-1.155 | 1.099  1.093-1.106 | <0.001 |
| **Total body** |  |  |  |  |  |
| Females  g/cm^2^ | Mean  (95%CI) | 1.144  1.140-1.147 | 1.160  1.156-1.163 | 1.207  1.203-1.210 | <0.001 |
| Males  g/cm^2^ | Mean  (95%CI) | 1.182  1.176-1.187 | 1.227  1.222-1.232 | 1.313  1.307-1.318 | <0.001 |

Data are shown as mean and 95%CI adjusted for weight. CI=confidence intervals. Main effect of time from repeated measured ANOVA. ANOVA=Univeriate analysis of variance.
